# Development and Validation of a Prognostic Signature Associated With Tumor Microenvironment Based on Autophagy-Related lncRNA Analysis in Hepatocellular Carcinoma

**DOI:** 10.3389/fmed.2021.762570

**Published:** 2021-12-14

**Authors:** Yan Deng, Feng Zhang, Zhen-Gang Sun, Shuai Wang

**Affiliations:** ^1^Department of Hepatobiliary Surgery, Jing Zhou Central Hospital, The Second Clinical Medical College, Yangtze University, Jing Zhou, China; ^2^Department of Ophthalmology, Jing Zhou Central Hospital, The Second Clinical Medical College, Yangtze University, Jing Zhou, China

**Keywords:** hepatocellular carcinoma, long non-coding RNA, autophagy, prognostic signature, TCGA, ICGC

## Abstract

**Objective:** The present study aimed to establish a prognostic signature based on the autophagy-related long non-coding RNAs (lncRNAs) analysis in patients with hepatocellular carcinoma (HCC).

**Methods:** Patients with HCC from The Cancer Genome Atlas (TCGA) were taken as the training cohort, and patients from the International Cancer Genome Consortium (ICGC) were treated as the validation cohort. Autophagy-related lncRNAs were obtained *via* a co-expression network analysis. According to univariate and multivariate analyses, a multigene prognostic signature was constructed in the training cohort. The predictive power of the signature was confirmed in both cohorts. The detailed functions were investigated using functional analysis. The single-sample gene set enrichment analysis (ssGSEA) score was used to evaluate the tumor microenvironment. The expression levels of immunotherapy and targeted therapy targets between the two risk groups were compared. Finally, a nomogram was constructed by integrating clinicopathological parameters with independently predictive value and the risk score.

**Results:** Four autophagy-related lncRNAs were identified to establish a prognostic signature, which separated patients into high- and low-risk groups. Survival analysis showed that patients in the high-risk group had a shorter survival time in both cohorts. A time-independent receiver-operating characteristic (ROC) curve and principal component analysis (PCA) confirmed that the prognostic signature had a robust predictive power and reliability in both cohorts. Functional analysis indicated that the expressed genes in the high-risk group are mainly enriched in autophagy- and cancer-related pathways. ssGSEA revealed that the different risk groups were associated with the tumor microenvironment. Moreover, the different risk groups had positive correlations with the expressions of specific mutant genes. Multivariate analysis showed that the risk score also exhibited excellent predictive power irrespective of clinicopathological characteristics in both cohorts. A nomogram was established. The nomogram showed good discrimination, with Harrell's concordance index (C-index) of 0.739 and good calibration.

**Conclusion:** The four autophagy-related lncRNAs could be used as biological biomarkers and therapeutic targets. The prognostic signature and nomogram might aid clinicians in individual treatment optimization and clinical decision-making for patients with HCC.

## Introduction

Hepatocellular carcinoma (HCC) is one of the fatal tumors occurring worldwide due to its aggressive biological behavior, rapidly increasing frequency, and high mortality (1, 2). Undoubtedly, the most difficult challenges that most clinicians face are early diagnosis and surgical intervention (3). Despite significant improvements in diagnosis and multimodal therapies, the survival benefit remains limited, owning to high heterogeneity (4–6). Hence, reliable predictive and prognostic biomarkers should be discovered to improve risk prediction ability and guide individualized therapy.

Autophagy is a multistep lysosomal degradation system that facilitates metabolic adaptability and nutrition cycling. These are biological processes that keep cell functioning properly (7–9). Autophagy has also been implicated in a variety of diseases, including cancer (10). However, the roles of autophagy in cancer are bilateral. On one hand, autophagy could offer the essential circulating metabolic substrates and enzymes to respond to various adverse circumstances; on the other hand, inappropriate autophagy enables malignant cells to proliferate rapidly, especially in advanced cancer (11, 12). Many studies have looked into a novel possible target therapy by investigating autophagy mechanisms (13, 14).

Long non-coding RNA (lncRNA) is a class of newly found RNA transcripts that cannot code for proteins. It usually has more than 200 nucleotides (15). By controlling transcriptionally or post-transcriptionally biological processes such as autophagy, an increasing number of lncRNAs have been linked to various physiological and physiological progress, including gene expression regulation, RNA decay, microRNA regulation, and protein folding (16, 17). Accumulating evidence suggested that lncRNAs could inhibit or activate the autophagy process through altering autophagy-related genes or pathways (18, 19).

With rapid advances in the RNA-sequencing technology, the potential for utilizing a lncRNA as a biomarker to aid the cancer detection, treatment, or prognosis has been gradually revealed (20). Using a comprehensive analysis of microarray data from The Cancer Genome Atlas (TCGA) and International Cancer Genome Consortium (ICGC) databases, the current study aimed to establish an autophagy-related lncRNA prognostic signature and a prognostic nomogram to predict the clinical outcome of patients with HCC.

## Materials and Methods

### Patient Data Acquisition

RNA-sequencing of patients with HCC and accompanying clinical data were downloaded from the TCGA (https://portal.gdc.cancer.gov/) and ICGC (https://icgc.org/). Patients with a follow-up duration of <1 month were excluded for survival analysis. The training group consisted of 343 patients with HCC from the TCGA database, and the clinical data were shown in Supplementary Excel S1. At the same time, the validation group consisted of 230 patients with HCC from the ICGC database. The clinical information is shown in Supplementary Excel S2.

Due to the collection of all the data directly from public databases, no protocol was required from the ethical committee.

### Autophagy-Related lncRNAs Screening

A total of 232 autophagy-related genes were obtained from the Human Autophagy Database (HADb, http://autophagy.lu/clustering/index.html). Then, the expression levels of these autophagy-related genes were retrieved from the TCGA and ICGC data sets.

The co-expression network between the expression of lncRNAs and autophagy-associated genes was investigated. LncRNAs with a correlation coefficient |*R*| > 0.5 and *p* < 0.050 were considered to be autophagy-related lncRNAs.

The lncRNA–mRNA co-expression network was constructed to explore the relationships between the autophagy-related lncRNAs and their mRNA counterparts. Cytoscape software (version 3.7.2) was used to visualize the co-expression network. Sankey plot was utilized to reveal the detailed relationships by the R studio software using the “ggalluvial” R package.

### Construction of an Autophagy-Related lncRNA Signature

The “survival” R package performed the Kaplan–Meier (KM) method and univariate Cox regression analysis to screen out prognostic autophagy-related lncRNAs with both significant values of *p* < 0.050 in the training cohort. Then, among these nominated autophagy-related lncRNAs, the multivariate Cox regression analysis was employed by the “survival” R package to assess their contributions as prognostic factors. The lowest Akaike information criterion (AIC) value was used to find the best autophagy-related lncRNAs. Subsequently, the risk score was established by the multiplication of the sum of the coefficients using autophagy-related lncRNAs expressions.

### Evaluation and Validation of the Prognostic Signature

A risk score was assigned to each patient with HCC. Based on the median value of their risk scores, all patients were classified into high- (high-risk score) and low-risk (low-risk score) groups. The prognosis of the two groups was compared using the KM survival curve, and the difference was assessed using a two-sided log-rank test. Time-dependent receiver-operating characteristic (ROC) curve analysis was performed using the “survival,” “survminer,” and “timeROC” R packages to evaluate the specificity and sensitivity of the prognostic signature. The prognosis accuracy was measured by the area under the ROC curve (AUC), a measure of discrimination. AUC ranges from 0.5 (little predictive power) to 1 (perfect prediction). Principal component analysis (PCA) was performed using the “ggplot2” R package to explore distinguishability. Following that, the distribution of patient's risk scores and scatter dots plot were depicted to visualize the detailed correlations of dead states with risk scores.

The subgroup survival analysis stratified by clinicopathological variables was conducted to evaluate the prognostic signature's accuracy across multiple cohorts.

### Functional Analysis

The Gene Ontology (GO) and the Kyoto Gene and Genomic Encyclopedia (KEGG) were used to enhance the potential functional pathways and categories based on co-expressed genes of autophagy-related lncRNAs. Significant values of *p* and *q* were defined as < 0.050. GO and KEGG analyses were conducted by applying the “org.Hs.eg.db,” “colorspace,” “stringi,” “ggplot2,” “dose,” “clusterProfiler,” and “enrichplot” R packages.

The gene set enrichment analysis (GSEA) was utilized to interpret the functional enrichment of gene expression data. This method derives its function by analyzing gene sets to determine whether the gene set shows a statistically significant difference between the two biological states. Within the “Molecular Signatures Database” of c2.cp.kegg. v6.2. Symbols by GSEA with a Java software, underlying mechanisms were studied. The random sample permutation number was set as 1,000, and the significance threshold *p* < 0.050.

### Evaluation of Immune Cell Infiltration Level, Tumor Purity, and Stromal Content

ESTIMATE was performed to investigate the immune cell infiltration level (immune score), tumor purity, and stromal content for each sample (21). The single-sample GSEA (ssGSEA) score was used to quantify the activity and enrichment level of immune cell types, functions, and pathways applying the “limma,” “GSVA,” and “GSEABase” R packages to all samples. The “pheatmap” R package exhibited heatmap results. The Spearman correlation was utilized to identify the correlations between risk score and tumor purity as well as stromal score. The Wilcoxon rank-sum test was performed to assess the difference between high- and low-risk groups, and the result was exhibited by the “ggpubr” R package.

### Correlation of the Prognostic Signature With Targets of Targeted Therapy and Immunotherapy

For the treatment of malignant tumors, targeted therapy and immunotherapy have become practical approaches. Now, the expression levels of immunotherapy and targeted therapy target genes between high- and low-risk groups were compared. We sought to predict therapeutic effectiveness using our risk score. The therapy targets were given as follows: programmed cell death 1 (PD-1, also known as PDCD1), vascular Endothelial Growth Factor Receptor (VEGFR1, also known as FLT1), Fms-like tyrosine kinase 3 (FLT3), VEGFR 3 (VEGFR3, also known as FLT4), platelet-derived growth factor receptor alpha (PDGFRA), platelet-derived growth factor receptor beta (PDGFRB), KIT proto-oncogene (KIT), ret proto-oncogene (RET), and MET proto-oncogene (MET), programmed cell death ligand 1 (PD-L1, also known as CD274), and mammalian target of rapamycin (mTOR). These correlations were drawn using the “ggpubr” R package, and the difference was evaluated by a Wilcoxon rank-sum test.

### LncRNA Expression Analysis

First, the raw data of the GSE101728 and GSE62232D data sets were freely downloaded from the Gene Expression Omnibus (GEO) database. GSE101728 data set contained seven pairs of tumor and normal tissues. There were 81 tumor and 10 normal tissues in the GSE62232D data set. Differential analysis between the targeted lncRNAs expressions was further investigated in the tumor and normal tissues. The different expressions of these lncRNAs were further explored in the TCGA and ICGC databases. Differential analysis was visualized using the “ggpubr” R package. Finally, these targeted lncRNA expression levels were compared based on previous original studies' quantitative real-time PCR results (22–31).

### Independence of the Prognostic Signature From Clinicopathological Parameters

Univariate and multivariate Cox proportional hazard regression analyses were performed by the “survival” R package to see if the predictive power of the prognostic signature was independent of clinicopathological parameters in both cohorts.

### Establishment and Evaluation of a Nomogram for Survival Prediction

To accurately predict the 1-, 3-, and 5-year overall survival (OS) probability, a prognostic nomogram was constructed by integrating clinicopathological parameters with independently predictive value and the risk score. Harrell's concordance index (C-index) was performed to evaluate the predictive accuracy. C-index ranges from 0.5 (no predictive power) to 1 (perfect prediction). Calibration plots were used to assess the nomogram's performance characteristics. Each patient would get the total points from the nomogram, namely Nomo-score, and patients were classified into three risk groups using the tertiles of Nomo-scores as the cut-off values. The performance of the nomogram was further investigated *via* a KM curve analysis.

### Statistical Analysis

All statistical analyses and figure generations were performed by the Perl programming language (version 5.30.2, http://www.perl.org) or R software (version 4.0.2, https://www.r-project.org/). A co-expression network was constructed using Cytoscape 3.6.1. A two-sided value of *p* < 0.050 was deemed statistically significant.

## Results

### Identification of Autophagy-Related lncRNAs in Tissue Samples of a Patient With HCC

The expression levels of 232 autophagy-related genes were extracted from the TCGA and ICGC database. Subsequently, the co-expression network analysis identified autophagy-related lncRNAs with |*R*| > 0.5 and *p* < 0.050 as the selection criteria. Finally, autophagy-related lncRNAs from the two cohorts were intersected, yielding 19 autophagy-related lncRNAs.

### Construction and Validation of an Autophagy-Related lncRNAs Prognostic Signature in the Training Cohort

**Figure 1:**
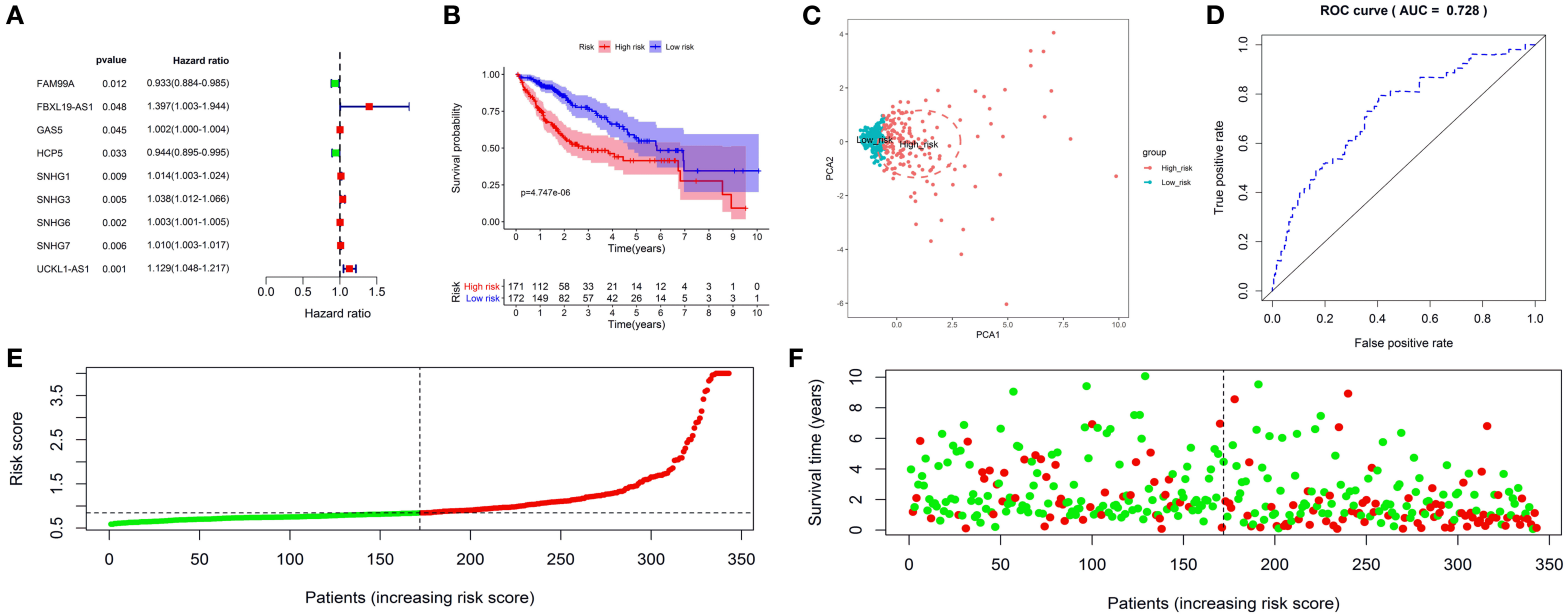
Construction and validation of an autophagy-related long non-coding RNA (lncRNA) prognostic signature in the training cohort. The forest map showed that 9 autophagy-related lncRNAs might be correlated with overall survival based on the Kaplan–Meier (KM) method and univariate Cox regression analysis **(A)**. The KM survival analysis showed that patients in the high-risk group had a shorter overall survival time **(B)**. Principal component analysis (PCA) showed that the high- and low-risk patients were located in two distinct distribution clusters; the red dots represented high-risk patients, whereas the blue dots represented low-risk patients **(C)**. The time-dependent receiver-operating characteristic (ROC) curve showed that the area under the ROC curve (AUC) value for the prognostic signature was 0.728 **(D)**. The distribution of risk scores between low- and high-risk groups; The red dots represented high-risk patients, whereas the green dots represented low-risk patients **(E)**. The scatter plot showed the relationship between the risk score and survival time; the red dots represented high-risk patients, whereas the green dots represented low-risk patients **(F)**.

**Figure 2:**
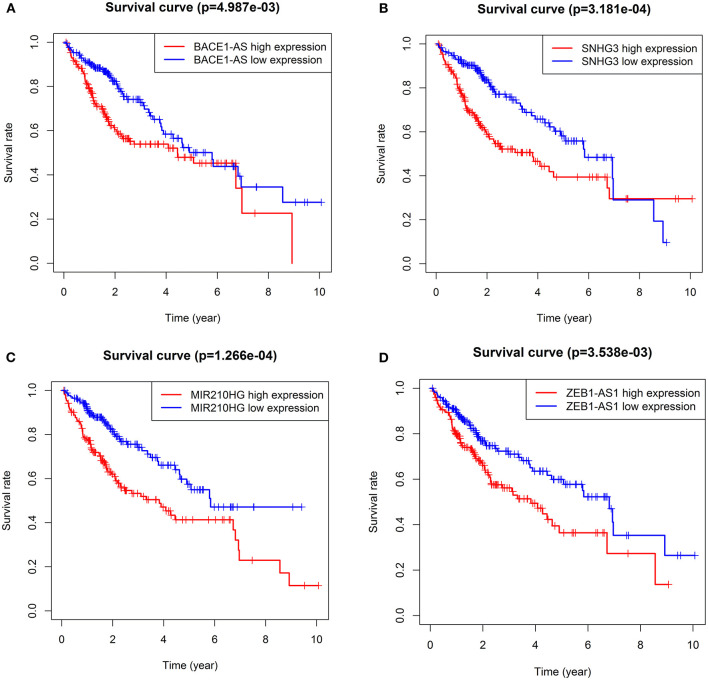
KM survival curves showed the four autophagy-related lncRNA risk factors for hepatocellular carcinoma (HCC). BACE1-AS **(A)**, SNHG3 **(B)**, MIR210HG **(C)**, and ZEB1-AS1 **(D)**.

Survival analysis showed that nine autophagy-related lncRNAs significantly correlated with OS (Figure 1A). Subsequently, a multivariate analysis revealed that four of nine autophagy-related lncRNAs were excellent candidates for constructing a prognostic signature. The candidates were BACE1-AS, SNHG3, MIR210HG, and ZEB1-AS1. The four lncRNAs have been confirmed to be risk factors (Figure 2). Following that, these autophagy-related lncRNAs were utilized to construct the following prognostic signature: risk score = (0.142 × the expression level of BACE1-AS) + (0.032 × the expression level of SNHG3) + (0.067 × the expression level of MIR210HG) + (0.112 × the expression level of ZEB1-AS1).

A risk score was assigned to each subject. The median risk score was the cut-off point to separate patients into high- or low-risk groups. The KM survival analysis revealed that the high-risk group had a shorter OS than the low-risk group (*p* < 0.050) (Figure 1B). Patients in the high-risk group had 1-, 3-, and 5-year survival rates of 75.60, 49.90, and 41.50%, whereas patients in the low-risk group had 1-, 3-, and 5-year survival rates of 93.40, 76.30, and 57.00%. The PCA analysis revealed that the high- and low-risk patients were located in the two distinct distribution clusters (Figure 1C). Time-dependent ROC curve analysis further showed that the AUC value for the prognostic signature was 0.728 (Figure 1D). The distribution ranking of patients' risk scores in different groups was shown in Figure 1E. The correlations of dead status with the risk score was shown using the scatter dots plot (Figure 1F). These results demonstrated that patients with a higher risk score suffered from a shorter survival duration and a poorer survival rate.

### Validation of the Prognostic Signature in the Validation Cohort

**Figure 3:**
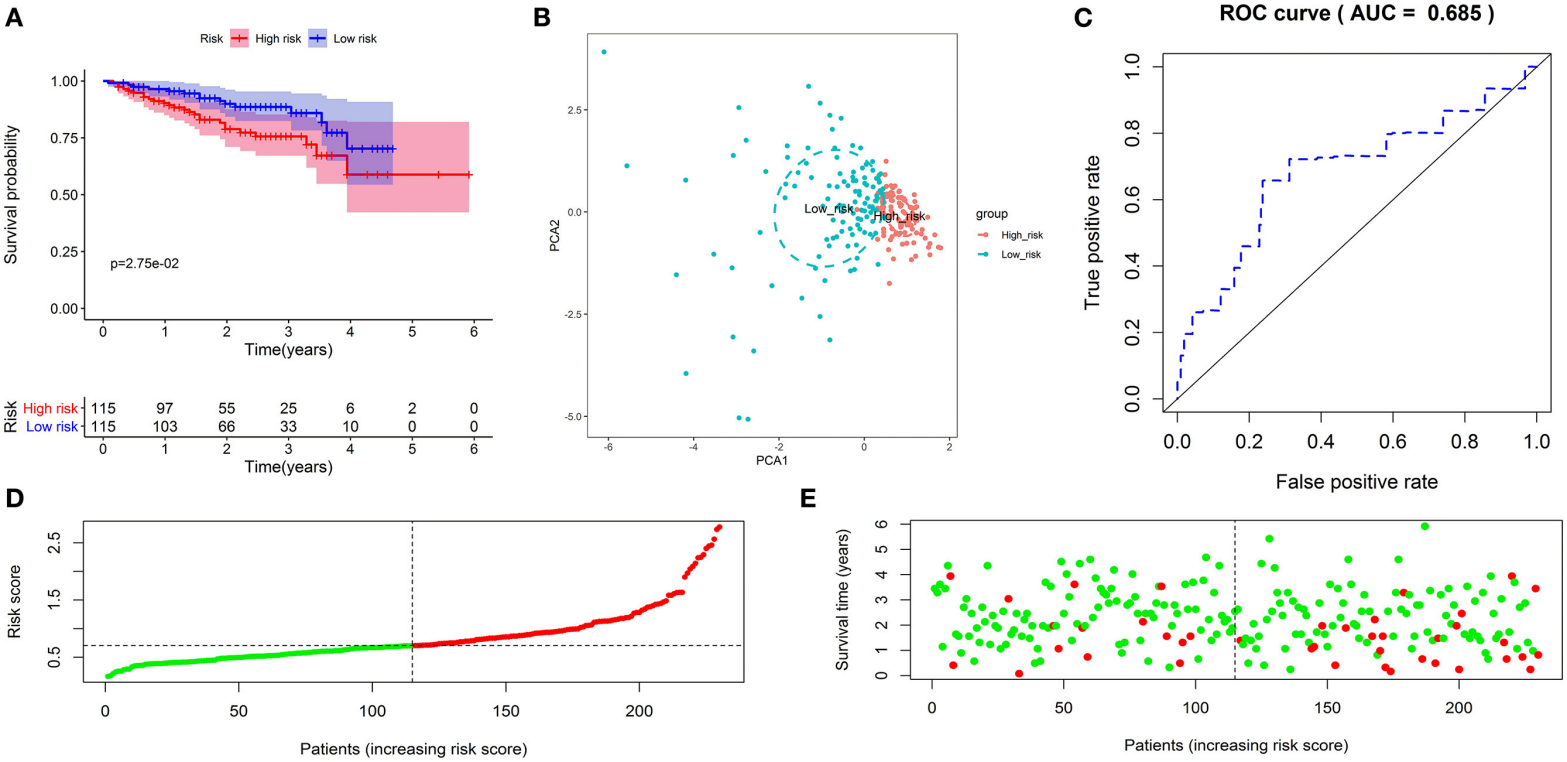
Validation of the autophagy-related lncRNA prognostic signature in the validation cohort. KM survival curves showed that patients in the high-risk group had significantly poorer overall survival **(A)**. Principal component analysis showed that patients with different risks were significantly divided into two clusters; the red dots represented high-risk patients, whereas the blue dots represented low-risk patients **(B)**. The time-dependent ROC curve showed that the AUC value for the prognostic signature was 0.685 **(C)**. The distribution of risk scores between low- and high-risk groups; The red dots represented high-risk patients, whereas the green dots represented low-risk patients **(D)**. The scatter plot showed the relationship between the risk score and survival time; The red dots represented high-risk patients, whereas the green dots represented low-risk patients **(E)**.

Subsequently, we further investigate the predictive value of the prognostic signature in the validation cohort. The same formula calculated the risk score and separated patients into low- or high-risk groups. As expected, the KM curve demonstrated that patients in the high-risk group had a shorter survival time (Figure 3A). PCA showed that patients of different risk groups were significantly split into two clusters (Figure 3B). Moreover, a time-dependent ROC curve was generated to validate the prognosis accuracy (AUC = 0.685), confirming the robust prediction of the signature (Figure 3C). Figures 3D,E depict the distribution of risk scores with survival status. These findings supported the hypothesis that the prognostic signature could reliably predict the prognosis of patients with HCC.

### Construction of the Autophagy-Associated lncRNA–mRNA Co-expression Network and Functional Enrichment Analysis

**Figure 4:**
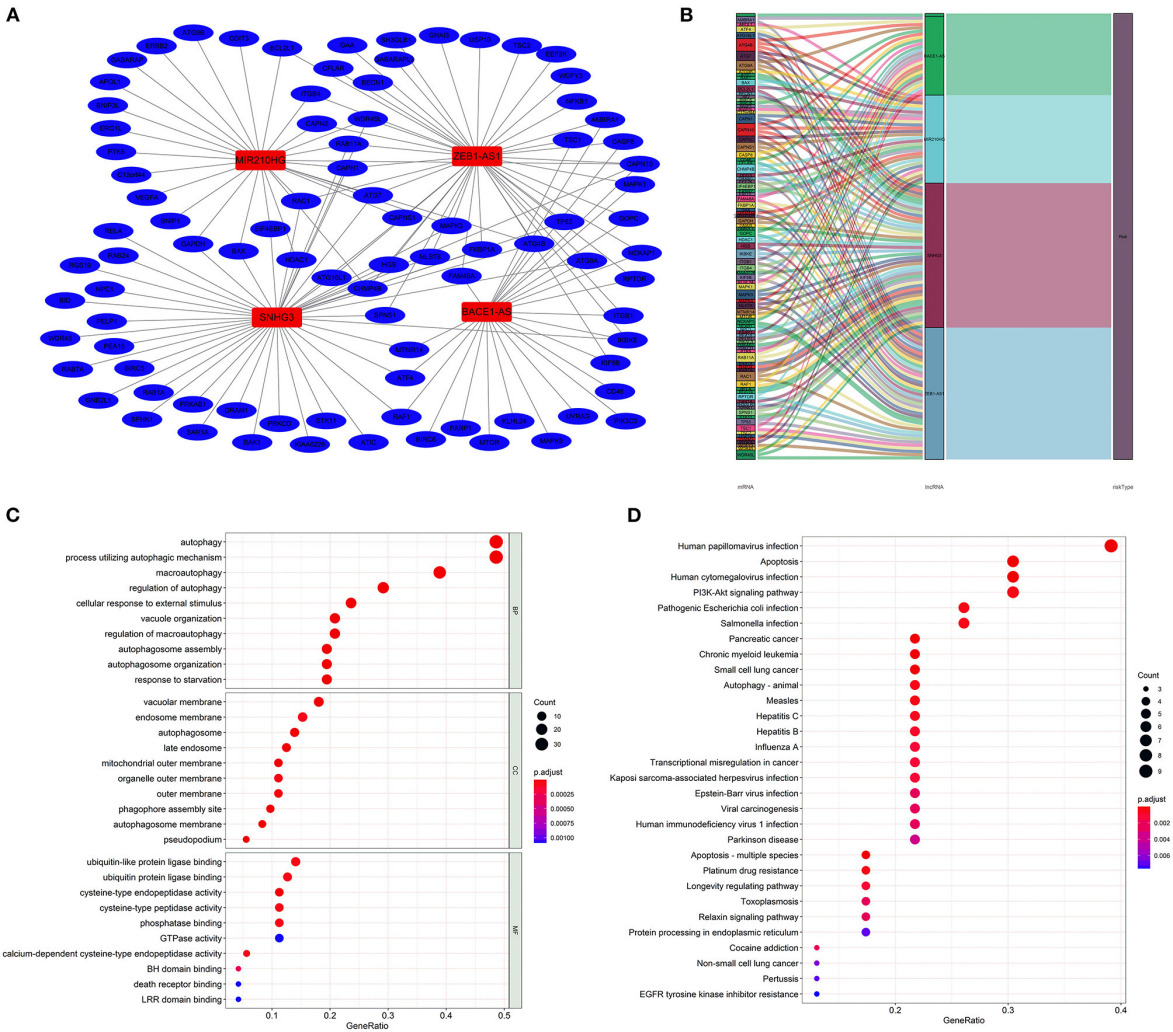
Construction of the autophagy-associated lncRNA–mRNA co-expression network and functional enrichment analysis. A network of the four lncRNAs with co-expressed autophagy-related genes. Red nodes represented lncRNAs, blue nodes represented mRNA, and each edge represented a co-expression relationship **(A)**. Sankey plot showed the detailed relationships of the four lncRNAs with autophagy-related genes and risk types **(B)**. Gene Ontology (GO) **(C)** and Kyoto Encyclopedia of Genes and Genomes **(D)** pathway analyses showed that these co-expressed genes were mainly correlated with autophagy and enriched in cancer-related pathways.

The lncRNA–mRNA co-expression network was constructed to probe potential functions. As shown in Figure 4A, the network contains 4 lncRNAs, 91 mRNAs, and 141 lncRNA–mRNA pairs. The detailed correlations of these lncRNAs with genes and risk types are also shown on the Sankey plot (Figure 4B). The GO and KEGG pathway analyses demonstrated that the genes encoded by these mRNAs were mainly correlated with autophagy and enriched in pathways in cancer (Figures 4C,D).

### Gene Set Enrichment Analysis

**Figure 5:**
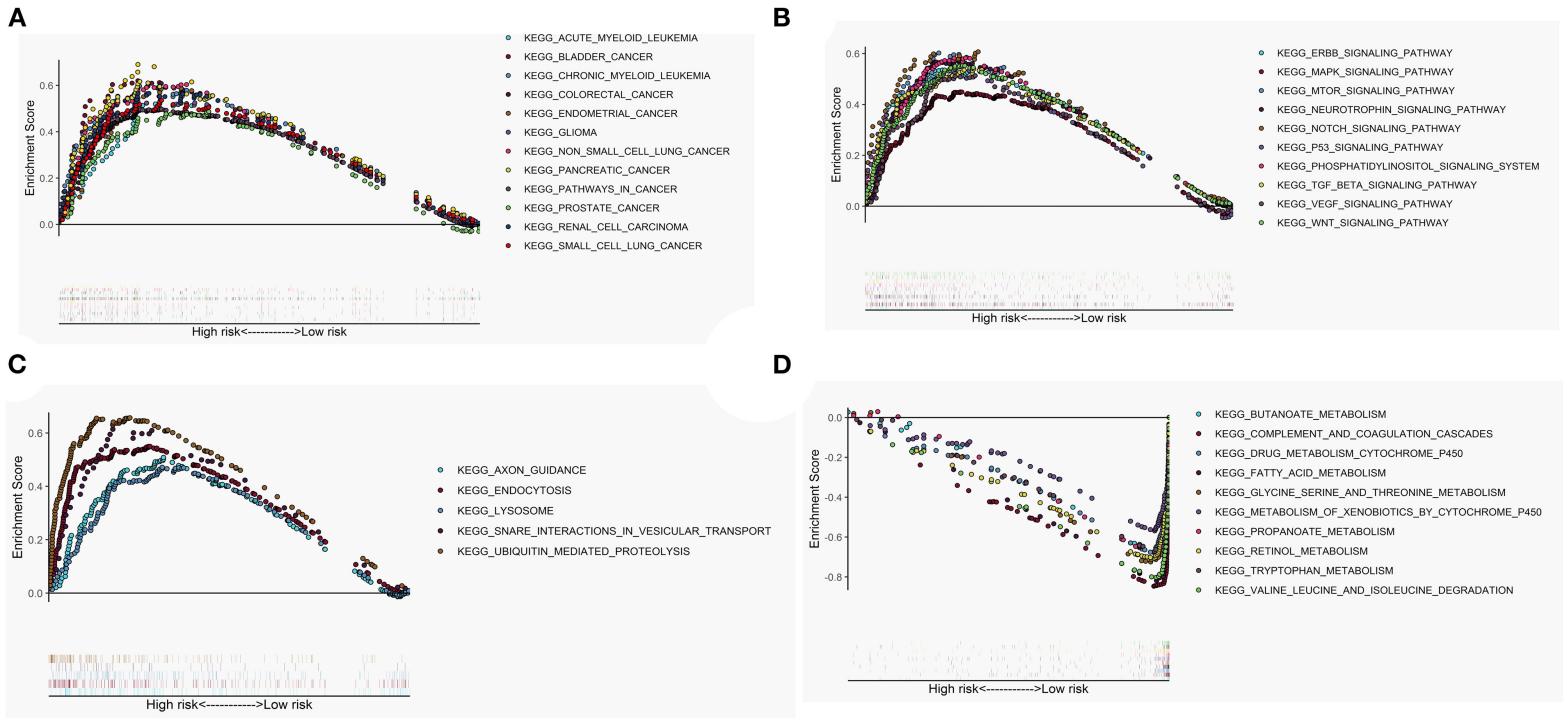
Gene set enrichment analysis (GSEA) between high- and low-risk groups. The altered gene sets in the high-risk group were mainly enriched in tumorigenesis **(A)**, cancer- and autophagy-related signaling pathways **(B)**, and physical actions of autophagy **(C)**. The protective pathways involved in metabolism were significantly enriched in the low-risk group **(D)**.

The GSEA performed a functional annotation. The GSEA results revealed that the altered gene sets in the high-risk group were directly involved in carcinogenesis and progression (Figure 5A). Besides, differentially expressed genes between the two risk groups were mainly enriched in the autophagy-associated and tumor-related pathways, including ERBB signaling pathway, MAPK signaling pathway, mTOR signaling pathway, VEGF signaling pathway, WNT signaling pathway, and P53 signaling pathway (Figure 5B). In addition, the altered expression genes in the high-risk group were discovered to be involved in autophagy's physical effects (Figure 5C). In contrast, the protective metabolic pathways were significantly enriched in the low-risk group (Figure 5D).

### Comparison of the Immune Activity and Tumor Microenvironment Between High- and Low-Risk Groups

**Figure 6:**
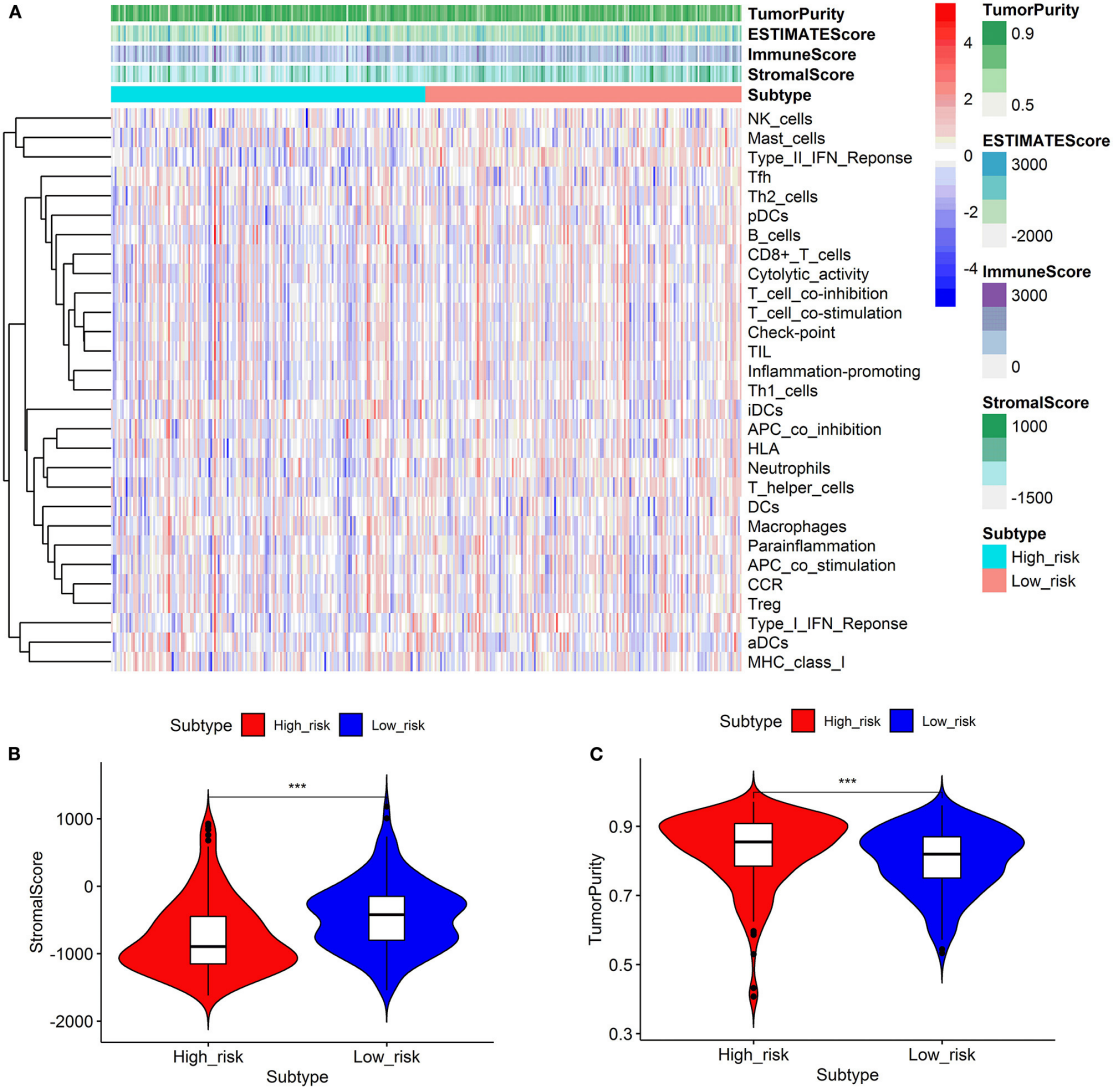
Comparison of the immune activity and tumor microenvironment between high- and low-risk groups. Hierarchical clustering showed that the low-risk group exhibited more immune cell infiltration than the high-risk group **(A)**. Comparison of stromal scores between the high- and low-risk groups **(B)**. Comparison of the tumor purity between high- and low-risk groups **(C)**. ***p < 0.001.

We looked at 29 immune-associated gene sets that represented various immune cell types, functions, and pathways. The activity and enrichment levels of immune cell types, functions, and pathways in each sample were measured using the ssGSEA score. Then, the enrichment scores were compared between low- and high-risk groups. Figure 6 showed that the low-risk group exhibited more significant immune cell infiltration than the high-risk group. Furthermore, the 13 immunological pathways in the low-risk group were more active than those in the high-risk group. When comparing the tumor purity and stromal scores between the two risk groups, we discovered that the stromal score was significantly higher in the low-risk group. In contrast, the tumor purity trended in the opposite direction, with tumor purity increasing from low risk to high risk (Kruskal–Wallis test, *p* < 0.001).

### Effectiveness Prediction of Immunotherapy and Targeted Therapy With the Prognostic Signature

**Figure 7:**
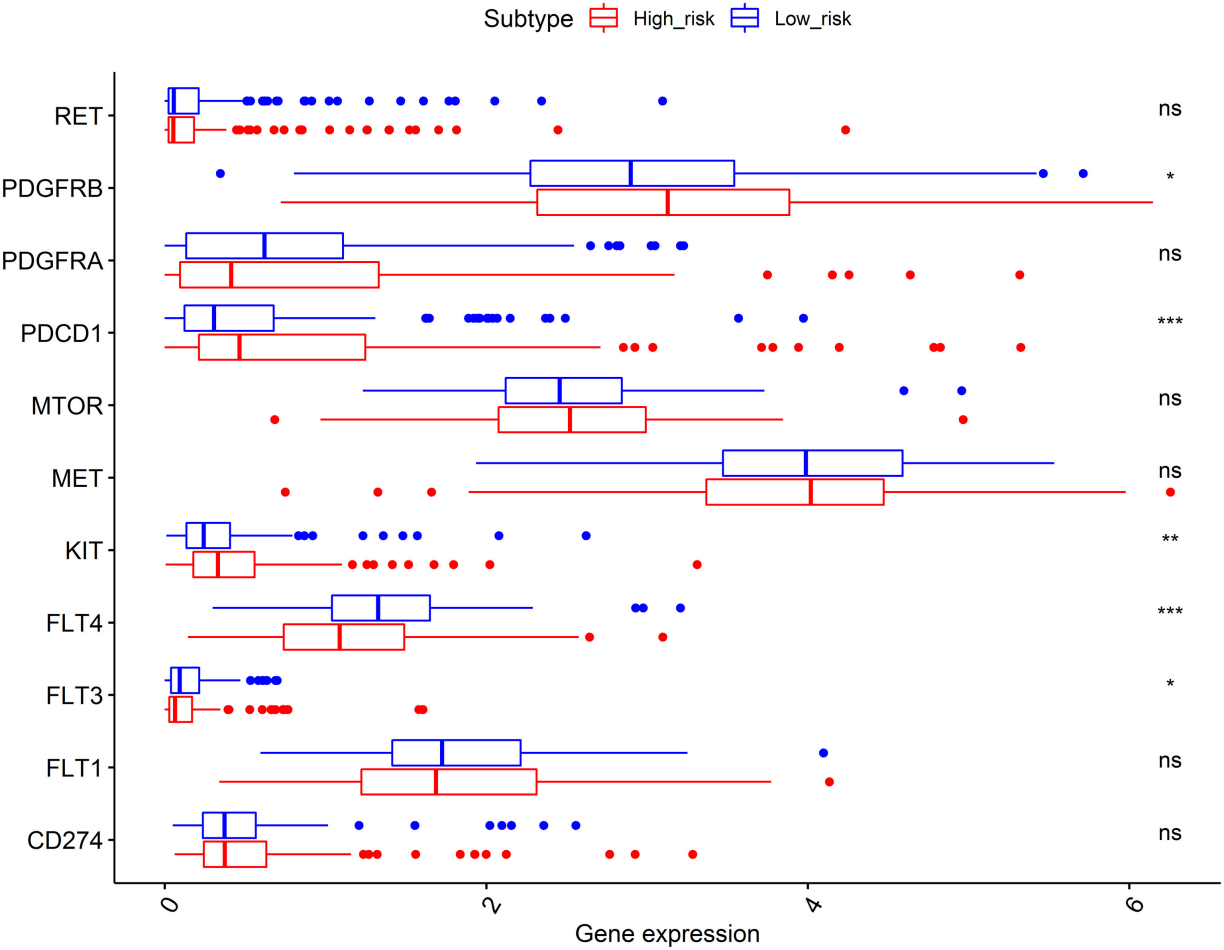
Comparisons of the expression levels of immunotherapy and targeted therapy target genes between high- and low-risk subgroups. The platelet-derived growth factor receptor beta (PDGFRB), programmed cell death 1 (PDCD1), and KIT proto-oncogene (KIT) expression levels were higher in the high-risk group, while the FLT3 and FLT4 expressions were higher in the low-risk group. *p < 0.050, **p < 0.010, and ***p < 0.001; ns, no significance.

As shown in Figure 7, the expression levels of immunotherapy and targeted therapy target genes were compared between high- and low-risk categories. The expression levels of PDGFRB, PDCD1, KIT, FLT3, and FLT4 between the two risk groups were significantly different. The PDGFRB, PDCD1, and KIT expression levels were higher in the high-risk group, while FLT3 and FLT4 expressions were higher in the low-risk group. Therefore, immunotherapy and targeted therapy medicines targeting PDGFRB, PDCD1, and KIT may be more effective in patients with HCC with higher risk scores.

### Expression Analysis of lncRNAs

**Figure 8:**
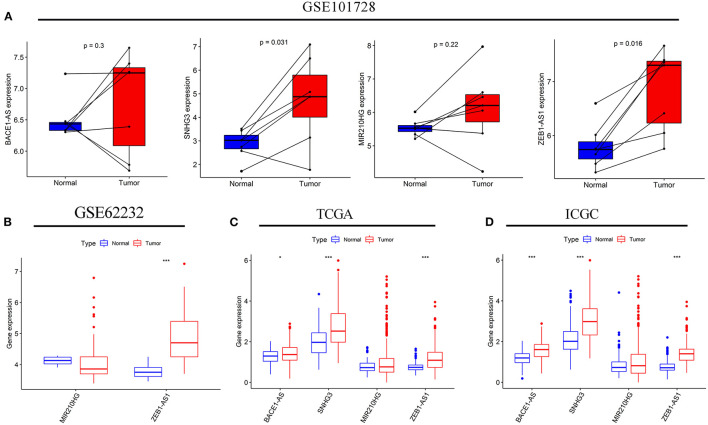
The expression levels of the four autophagy-related lncRNAs in HCC tissues and adjacent normal tissues. The expressions of the four lncRNAs in seven paired HCC and adjacent normal tissues from the GSE10728 data set **(A)**. Comparison of the MIR210HG and ZEB1-AS1 expressions between 10 normal tissues and 81 tumor tissues in the GSE62232 data set **(B)**. Comparison of the four autophagy-related lncRNAs expressions in normal and tumor tissues from the Cancer Genome Atlas (TCGA) **(C)** and International Cancer Genome Consortium (ICGC) **(D)** cohorts. *p < 0.050 and ***p < 0.001.

Subsequently, a difference in the expression of the four targeted lncRNAs was investigated between the tumor tissues and normal tissues. In the GSE101728 data set, the SNHG3 and ZEB1-AS1expression levels were higher in the tumor tissues, while the expression levels of BACE1-AS and MIR210HG showed no difference (Figure 8A). In the analysis results of the GSE62232 data set, ZEB1-AS1 was highly expressed in tumor tissues (Figure 8B). The analysis of HCC samples from the TCGA and ICGC databases both exhibited that BACE1-AS, SNHG3, and ZEB1-AS1 were highly expressed in the tumor tissue, and there was no significant difference in MIR210HG expression (Figures 8C,D). What is more, we have found ten original studies involving the differential expressions of the four targeted lncRNAs between normal and tumor tissues. Interestingly, the lncRNAs expression levels in HCC tumor tissues were significantly higher than those in the normal control group in each study (Supplementary Figure S1).

### Correlation Analysis of the Autophagy-Related lncRNA Prognostic Signature With Clinicopathological Features

Correlation analysis was done to investigate the clinical value of the prognostic signature in different subgroups stratified by the clinicopathological characteristics of the patients. As shown in Table 1, patients with high-risk scores were prone to be found in those with greater creatinine or Alpha-fetoprotein (AFP). The increasing risk score appeared to be highly connected with advanced T and TNM stages, suggesting that the prognostic signature may be considerably associated with HCC progression.

**Table 1:** Clinical value of the autophagy-related lncRNA prognostic signature for HCC.

**Characteristics**	**Group**	**Risk score**		
		***N***	**Mean**	***P*** **value**
Age (years)	<60	157	1.154	0.787
	≥60	186	1.183	
Gender	Male	233	1.114	0.120
	Female	110	1.289	
Alcohol consumption	Present	109	1.106	0.482
	Absent	218	1.185	
Liver cirrhosis	Present	131	1.254	0.353
	Absent	65	1.112	
Family history	Present	104	1.063	0.165
	Absent	194	1.216	
Histological grade	G1+G2	211	1.159	0.699
	G3+G4	138	1.202	
Albumin (g/dl)	<4.0	128	1.055	0.633
	≥4.0	153	1.105	
Creatinine (mg/dl)	<1.1	189	0.926	**0.005**
	≥1.1	92	1.169	
BMI (kg/cm2)	<25	143	1.163	0.776
	≥25	152	1.196	
AFP (ng/ml)	≤ 200	187	0.977	**0.006**
	>200	73	1.408	
ECOG	=0	156	1.094	0.196
	>0	117	1.253	
T stage	I+II	252	1.042	**0.002**
	III+IV	88	1.551	
TNM stage	I+II	238	1.025	**0.001**
	III+IV	83	1.576	

*Bold font represents P < 0.05 and the relevant variables are statistically significant*.

*HCC, hepatocellular carcinoma; HBV, hepatitis B virus; HCV, hepatitis C virus; AFP, alpha-fetoprotein; BMI, body mass index; ECOG, eastern cooperative oncology group; TNM, tumor node metastasis; SD, standard deviation*.

### Prognostic Value of the Autophagy-Related lncRNAs Signature Among Different Subgroups

Subgroup analysis was conducted to investigate the prognostic value of the autophagy-related lncRNA signature among different subgroups stratified by clinicopathological variables. As indicated in Table 2, the prognostic signature performs better in male patients without liver cirrhosis and family history, whereas obese individuals in poor physical condition may benefit more from the prognostic signature. The prognostic signature seemed to be more applicable to patients with relatively lower serum AFP, albumin, and creatinine levels in terms of laboratory index. Besides, the prognostic signature showed excellent predictive power independent of various clinicopathological features such as gender, age, alcohol consumption history, tumor stage, and histological grade.

**Table 2:** Prognostic value of the autophagy-related lncRNA prognostic signature in different subgroups stratified by clinicopathological variables.

**Characteristics**	**Group**	**Low/High**	**HR (95% CI)**	***P*** **value**
Overall		172/171	2.249 (1.559–3.256)	**<0.001**
Age	<60	76/81	2.832 (1.586–5.056)	**<0.001**
	≥60	95/91	1.754 (1.079–2.853)	**0.023**
Gender	Male	124/109	3.125 (1.925–5.072)	**<0.001**
	Female	47/63	1.290 (0.7285–2.285)	0.383
Alcohol consumption	Present	60/49	3.718 (1.921–7.194)	**<0.001**
	Absent	106/112	1.794 (1.136–2.833)	**0.012**
Liver cirrhosis	Present	26/39	2.108 (1.010–4.396)	**0.047**
	Absent	70/67	1.922 (0.962–3.836)	0.064
Family history	Present	63/41	1.828 (1.019–3.277)	0.043
	Absent	83/111	2.944 (1.688–5.133)	**<0.001**
Histological grade	G1+G2	102/109	2.154 (1.338–3.466)	**0.002**
	G3+G4	66/61	2.549 (1.411–4.607)	**0.002**
Albumin (g/dl)	<4.0	76/52	1.908 (1.040–3.501)	**0.037**
	≥4.0	75/78	1.644 (1.893–3.025)	0.110
Creatinine (mg/dl)	<1.1	98/91	1.721 (1.034–2.862)	**0.037**
	≥1.1	53/39	1.736 (0.825–3.656)	0.147
BMI (kg/cm2)	<25	70/73	1.670 (0.917–3.040)	0.093
	≥25	77/75	3.805 (2.105–6.787)	**<0.001**
AFP (ng/ml)	≤ 200	116/171	1.985 (1.156–3.408)	**0.013**
	>200	21/52	2.041 (0.757–5.506)	0.159
ECOG	=0	91/65	1.878 (0.924–3.817)	0.081
	>0	53/64	3.016 (1.679–5.416)	**<0.001**
T stage	I+II	140/112	2.064 (1.280–3.329)	**0.003**
	III+IV	29/59	1.926 (1.054–3.520)	**0.033**
TNM stage	I+II	135/103	1.915 (1.155–3.174)	**0.012**
	III+IV	26/57	2.025 (1.046–3.921)	**0.036**

*Bold font represents P < 0.050 and the relevant variables are statistically significant*.

*HBV, hepatitis B virus; HCV, hepatitis C virus; AFP, alpha-fetoprotein; BMI, body mass index; ECOG, eastern cooperative oncology group; TNM, tumor node metastasis; HR, hazard ratio*.

### Independence of the Prognostic Signature From Clinicopathological Parameters

**Figure 9:**
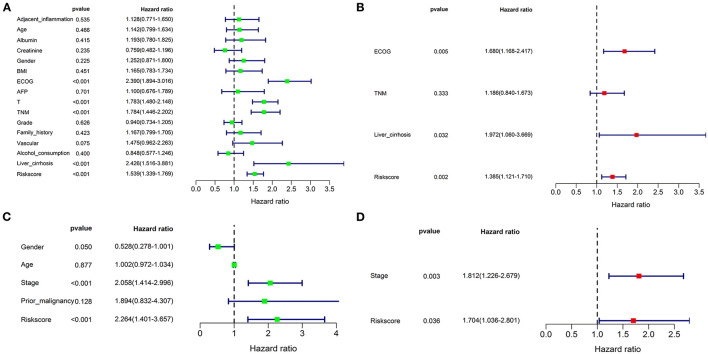
Univariate and multivariate Cox regression analyses for the risk score combined with the clinical characteristics. Univariate analysis for the TCGA **(A)** and ICGC **(C)** cohorts. These parameters with a prognostic value were enrolled into the multivariate Cox regression analysis. The result showed that the risk score was an independently prognostic indicator in TCGA **(B)** and ICGC **(D)** cohorts.

Univariate and multivariate Cox regression analyses were performed to assess whether the prognostic signature was a prognostic factor independent of clinicopathological features in both cohorts. As shown in Figure 9A, univariate analysis indicated that ECOG [HR: 2.390; 95% CI: 1.894–3.016; *p* < 0.001], TNM stage [HR: 1.784; 95% CI: 1.446–2.202; *p* < 0.001], liver cirrhosis [HR: 2.426; 95% CI: 1.516–3,881.; *p* < 0.001], and the risk score [HR: 1.539; 95% CI: 1.339–1.769; *p* < 0.001] were significantly correlated with OS in the training cohort. T stage was not enrolled in multivariate Cox regression modeling because the TNM stage was derived based on the T, N, and M stages. ECOG [HR: 1.680; 95% CI: 1.168–2.417; *p* = 0.005], liver cirrhosis [HR: 1.972; 95% CI: 1.060–3.669; *p* = 0.032], and the risk score [HR: 1.385; 95% CI: 1.121–1.710; *p* = 0.002] were ruled out as independently prognostic factors in multivariate analysis (Figure 9B). Besides, the risk score was also proven to be an independently prognostic factor in the validation cohort (Figures 9C,D).

### Establishment of a Nomogram for OS Prediction

**Figure 10:**
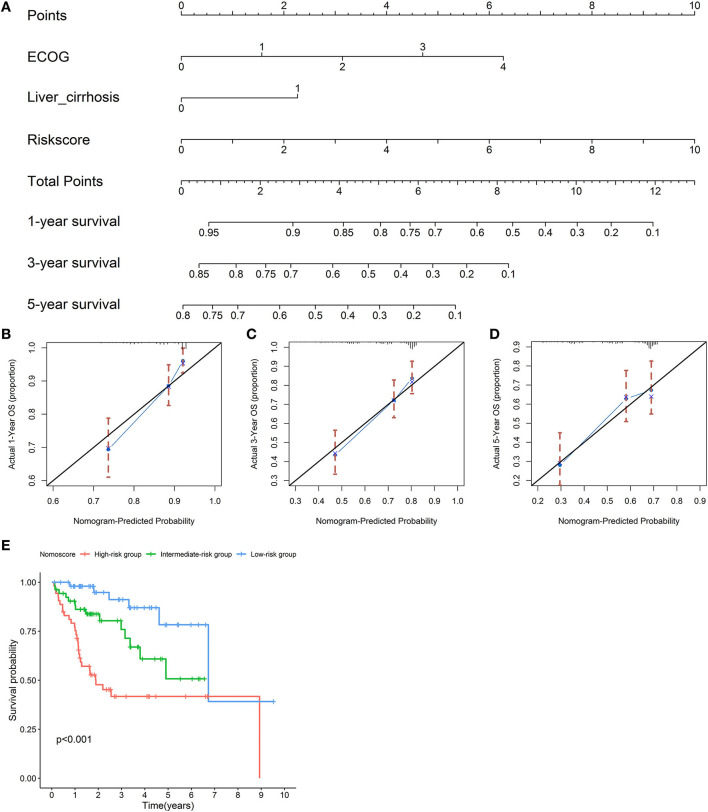
Establishment of a nomogram for overall survival (OS) prediction. A nomogram for predicting 1-, 3-, and 5-year OS was constructed based on three independent prognostic factors: the risk score, ECOG, and liver cirrhosis. The detailed 1-, 3-, and 5-year OS rates were displayed in the nomogram **(A)**. Calibration plots showed that the nomogram performed well in the predicted 1- **(B)**, 3- **(C)**, and 5-year **(D)** survival probabilities with an ideal model. The black line represents the “ideal” line of a perfect match between the predicted and observed survival. The blue line indicates the performance of the proposed nomogram. X-axis is the nomogram predicted probability of survival, and Y-axis is actual survival. Blue dots are subcohorts of the data set; red vertical bars represent a 95% CI. KM curves of three risk subgroups stratified by the tertiles of Nomo-scores showed the healthy performance of the nomogram **(E)**.

An OS nomogram was formulated based on three independently prognostic factors in the training cohort. Furthermore, the 1-, 3-, and 5-year OS rate was displayed in the nomogram (Figure 10A). The C-index value for OS prediction was 0.739. Calibration plots further identified that the nomogram performed well in predicted 1-, 3-, and 5-year survival probabilities with an ideal model, indicating that the nomogram was perfectly calibrated to predict OS at assessing the performance characteristics (Figures 10B–D). Each patient with complete clinical information on the ECOG score and liver cirrhosis (or not) would get the Nomo-score, and patients were classified into three risk categories based on the tertiles of Nomo-scores. The KM curve revealed significant variations across high-, intermediate-, and low-risk groups (*p* < 0.001) (Figure 10E).

## Discussion

Hepatocellular carcinoma is one of the most lethal and prevalent primary hepatic malignant neoplasms worldwide. Despite great improvements in diagnosis and multimodal therapies, the survival benefit remains limited due to high heterogeneity (32). Clinically, histological grade, tumor stage, molecular subtype, and serum indicator prognostic effects were evaluated (33). However, such clinicopathological characteristics were unable to provide predictive value, resulting in inaccurate prognosis judgment. According to the situation, certain high-risk patients may encounter tumor cell uncontrollable growth due to insufficient treatment, while low-risk patients may receive excessive treatment, resulting in long-term toxicity and morbidity. Therefore, reliable genetic signatures or biomarkers as prognostic predictors or therapeutic targets are of significance for HCC.

The overutilization of amino acids such as tryptophan, aerobic glycolysis, tricarboxylic acid (TCA) cycle, glutamine, arginine, defective mitochondrial bioenergetics, and oxidative stress phosphorylation are all involved in malignant cell progression and extinction (34, 35). Moreover, these energy metabolisms are dramatically associated with autophagy progress. Hence, knowing the specifics and direct links between autophagy and tumor progression could provide a solid foundation for creating drugs targeting these pathways and ultimately curing malignancies. Following decades of researches on prognostic gene biomarkers of tumor-related events such as microRNAs and mRNAs, lncRNAs have recently aroused much attention. The roles of lncRNA in carcinogenesis and malignant tumor growth have been gradually revealed. The prognostic value of lncRNA also has been extensively explored. However, there has yet to be a systematic method for identifying the autophagy-related lncRNAs signature that might be used to predict the prognosis of patients with HCC. Hence, developing an autophagy-related lncRNAs signature to predict the clinical outcome is critical for patients with HCC.

In the study, we used the expression profile of HCC patients' tumor tissue from the TCGA and ICGC databases to investigate the prognostic usefulness of autophagy-related lncRNAs and develop a prognostic signature. We first identified 19 autophagy-related lncRNAs based on the lncRNAs and autophagy-related gene co-expression network. After univariate and multivariate Cox regression analyses, four autophagy-related lncRNAs, including BACE1-AS, SNHG3, MIR210HG, and ZEB1-AS1, were selected to establish a prognostic signature. Each patient obtained a risk score. All patients were divided into high or low risks based on the median value of risk scores. We also discovered that patients with varied risks were considerably split into two groups, with the high-risk group having a shorter survival time. The ROC curve analysis further confirmed the prognostic accuracy of the signature. When the predictive value of the prognostic signature was investigated in the ICGC validation cohort, similar results were achieved. Univariate and multivariate regression models showed that the risk score showed excellent predictive power independent of all clinicopathological characteristics in both cohorts. Hence, the autophagy-related lncRNA prognostic signature showed powerful potential for clinical applications.

When associations of the risk score and clinicopathological characteristics were investigated, we found that the risk score was significantly related to advanced tumor and a higher level of serum AFP. The explanation supported the findings that improper autophagy contributed to a poor tumor microenvironment, allowing the malignant cell to proliferate, invade, and migrate quickly as the tumor advanced. These alterations might lead to a poor prognosis for patients with advanced cancers (14, 36). Subgroups analyses stratified by clinicopathological variables further verified the steadied predictive value of the prognostic signature.

The role of autophagy in cancer is debatable. As the understanding of autophagy continues to deepen, the role has been increasingly revealed. On one hand, autophagy could provide the essential circulating metabolic substrates and enzymes to respond to various poor circumstances such as tumor microenvironment; on the other hand, inappropriate autophagy also promotes malignant cell rapid growth, particularly in the advanced tumor. LncRNAs' roles have recently been discovered to mediate tumorigenesis, progression, metastasis, and treatment resistance by regulating genes or microRNAs. The present study identified four autophagy-related lncRNAs to establish a prognostic signature. Previous studies confirmed that BACE1-AS was significantly associated with the prognosis of patients with cancer (22, 23). BACE1-AS could also enhance autophagy-related neuronal damage *via* the miR-214-3p/ATG5 signaling axis in Alzheimer's disease (37). Additionally, the functions of SNHG3 in cancer have been steadily revealed. A growing body of evidence showed that SNHG3 appeared to influence tumor formation and progression by modulating autophagy-related microRNAs, genes, or pathways (24–26). MIR210HG (27, 38–40) and ZEB1-AS1 (28–31, 41, 42) have also been found to alter tumorigenesis, progression, and tumor metastasis, resulting in a poor prognosis for patients with cancer. Unquestionably, the established prognostic signature based on the four robust autophagy-related lncRNAs had a more excellent predictive value. Subsequently, we also identified the genes governed by the four autophagy-related lncRNAs and established a lncRNA–mRNA co-expression network. GO and KEGG functional enrichment analyses showed that these genes were mainly enriched in autophagy- and tumor-related signaling pathways.

The GSEA functional enrichment analysis showed that autophagy- and cancer-related pathways were overrepresented in the high-risk group. The altered gene sets in the high-risk group were discovered to be engaged in the autophagy-associated and tumor-related pathways as well as the autophagy's physical effects. At the same time, the protective pathways involved in various metabolisms were significantly enriched in the low-risk group. As a result, our findings added to the growing body of evidence showing that autophagy is a crucial regulator of oncogenesis and development. We also concluded that the four autophagy-related lncRNAs could be therapeutic targets. Moreover, we looked at immune cell infiltration and antitumor immunological activity between high- and low-risk groups. The results revealed that patients with low-risk scores had more immune cell infiltrations and antitumor immune activities, showing that the high-risk group's immune functions were overall impaired. The increasing antitumor immune activity could explain why patients with low risk had well clinical outcomes. Moreover, we found that the stromal score was greater in the low-risk group, whereas the tumor purity increased from the low- to the high-risk subgroup. The results further demonstrated that the poor prognosis might be due to an unbenefited tumor microenvironment. Currently, immunotherapy and targeted therapy are hot fields of investigation. The prognostic signature also revealed that the risk score was significantly associated with the effectiveness of immunotherapy and targeted therapies, thus validating the signature's prediction accuracy.

Nomogram is an effective and reliable clinical tool that can generate a probabilistic forecast for an individual patient. To improve prognosis prediction for patients with HCC, we constructed an OS nomogram based on independently prognostic factors. Calibration plots showed that the predicted 1-, 3-, and 5-year survival rates were comparable with the actual observation. A high C-index indicated robust discrimination, implying that it might function as a predive tool. However, more research is needed to confirm the prognostic signature in a larger number of patients and to reveal the potential molecular mechanisms of the four autophagy-related lncRNAs in HCC.

## Conclusion

In conclusion, although the autophagy-related lncRNAs prognostic signature was a promising tool for predicting the prognosis of patients with HCC, there is a need for further studies to evaluate the device. The prognostic signature might aid in better understanding the role of autophagy in carcinogenesis and progression. The four autophagy-related lncRNAs might be used as potential biomarkers and therapeutic targets for patients with HCC. The prognostic signature and nomogram could be used to stratify patients at risk, aiding clinicians in treatment optimization and clinical decision-making.

## Data Availability Statement

The original contributions presented in the study are included in the article/Supplementary Material, further inquiries can be directed to the corresponding author.

## Author Contributions

YD and FZ contributed to conception and design, the acquisition of data, the analysis and interpretation of data, and drafting the article. Z-GS took part in drafting the article or revising it critically for important intellectual content and gave final approval of the version to be published. SW contributed to conception and design, revising the article critically for important intellectual content, and gave final approval of the version to be published. All authors agreed to be accountable for all aspects of the work.

## Conflict of Interest

The authors declare that the research was conducted in the absence of any commercial or financial relationships that could be construed as a potential conflict of interest.

## Publisher's Note

All claims expressed in this article are solely those of the authors and do not necessarily represent those of their affiliated organizations, or those of the publisher, the editors and the reviewers. Any product that may be evaluated in this article, or claim that may be made by its manufacturer, is not guaranteed or endorsed by the publisher.

## Supplementary Material

Supplementary Table 1 A list of 232 autophagy genes.

Supplementary Excel S1 Clinical data of patients with hepatocellular carcinoma (HCC) from the Cancer Genome Atlas (TCGA) database.

Supplementary Excel S2 Clinical data of patients with HCC from the International Cancer Genome Consortium (ICGC) database.

Supplementary Figure S1 The expression levels of the four autophagy-related long non-coding RNAs (lncRNAs) in hepatocellular carcinoma (HCC) tissues and adjacent normal tissues were detected by RT-PCR in each previous study. BACE1-AS **(A–B)**, SNHG3 **(C–E)**, MIR210HG **(F)**, and ZEB1-AS1 **(G–J)**.

## Abbreviations

lncRNAs: long non-coding RNAs
TCGA: The Cancer Genome Atlas
ICGC: International Cancer Genome Consortium
HCC: hepatocellular carcinoma
HADb: Human Autophagy Database
KM: Kaplan–Meier
AIC: Akaike information criterion
ROC: receiver-operating characteristic
AUC: area under the ROC curve
C-index: Harrell's concordance index
GO: Gene Ontology
KEGG: Kyoto Gene and Genomic Encyclopedia
GSEA: Gene set enrichment analysis
OS: overall survival.
