# Supplementary material for: Development and Validation of a Prognostic Signature Associated With Tumor Microenvironment Based on Autophagy-Related lncRNA Analysis in Hepatocellular Carcinoma
Source: Front Med (Lausanne). 2021 Dec 14;8:762570. doi: 10.3389/fmed.2021.762570 (PMC8712323; doi:10.3389/fmed.2021.762570)
Supplement: Supplementary Table 1 — A list of 232 autophagy genes. [file Data_Sheet_1.DOCX]

Supplementary Table 1: A list of 232 autophagy-related genes

| Autophagy genes | | | | | | | |
| --- | --- | --- | --- | --- | --- | --- | --- |
| AMBRA1 | BID | CTSD | FOXO1 | KIAA0226 | NRG1 | RELA | ULK2 |
| APOL1 | BIRC5 | CTSL1 | FOXO3 | KIAA0652 | NRG2 | RGS19 | ULK3 |
| ARNT | BIRC6 | CX3CL1 | GAA | KIAA0831 | NRG3 | RHEB | USP10 |
| ARSA | BNIP1 | CXCR4 | GABARAP | KIF5B | P4HB | RPS6KB1 | UVRAG |
| ARSB | BNIP3 | DAPK1 | GABARAPL1 | KLHL24 | PARK2 | RPTOR | VAMP3 |
| ATF4 | BNIP3L | DAPK2 | GABARAPL2 | LAMP1 | PARP1 | SAR1A | VAMP7 |
| ATF6 | C12orf44 | DDIT3 | GAPDH | LAMP2 | PEA15 | SERPINA1 | VEGFA |
| ATG10 | C17orf88 | DIRAS3 | GNAI3 | MAP1LC3A | PELP1 | SESN2 | WDFY3 |
| ATG12 | CALCOCO2 | DLC1 | GNB2L1 | MAP1LC3B | PEX14 | SH3GLB1 | WDR45 |
| ATG16L1 | CAMKK2 | DNAJB1 | GOPC | MAP1LC3C | PEX3 | SIRT1 | WDR45L |
| ATG16L2 | CANX | DNAJB9 | GRID1 | MAP2K7 | PIK3C3 | SIRT2 | WIPI1 |
| ATG2A | CAPN1 | DRAM1 | GRID2 | MAPK1 | PIK3R4 | SPHK1 | WIPI2 |
| ATG2B | CAPN10 | EDEM1 | HDAC1 | MAPK3 | PINK1 | SPNS1 | ZFYVE1 |
| ATG3 | CAPN2 | EEF2 | HDAC6 | MAPK8 | PPP1R15A | SQSTM1 |  |
| ATG4A | CAPNS1 | EEF2K | HGS | MAPK8IP1 | PRKAB1 | ST13 |  |
| ATG4B | CASP1 | EGFR | HIF1A | MAPK9 | PRKAR1A | STK11 |  |
| ATG4C | CASP3 | EIF2AK2 | HSP90AB1 | MBTPS2 | PRKCD | Symbol |  |
| ATG4D | CASP4 | EIF2AK3 | HSPA5 | MLST8 | PRKCQ | TBK1 |  |
| ATG5 | CASP8 | EIF2S1 | HSPA8 | MTMR14 | PTEN | TM9SF1 |  |
| ATG7 | CCL2 | EIF4EBP1 | HSPB8 | MTOR | PTK6 | TMEM49 |  |
| ATG9A | CCR2 | EIF4G1 | IFNG | MYC | RAB11A | TMEM74 |  |
| ATG9B | CD46 | ERBB2 | IKBKB | NAF1 | RAB1A | TNFSF10 |  |
| ATIC | CDKN1A | ERN1 | IKBKE | NAMPT | RAB24 | TP53 |  |
| BAG1 | CDKN1B | ERO1L | IL24 | NBR1 | RAB33B | TP53INP2 |  |
| BAG3 | CDKN2A | FADD | IRGM | NCKAP1 | RAB5A | TP63 |  |
| BAK1 | CFLAR | FAM48A | ITGA3 | NFE2L2 | RAB7A | TP73 |  |
| BAX | CHMP2B | FAS | ITGA6 | NFKB1 | RAC1 | TSC1 |  |
| BCL2 | CHMP4B | FKBP1A | ITGB1 | NKX2-3 | RAF1 | TSC2 |  |
| BCL2L1 | CLN3 | FKBP1B | ITGB4 | NLRC4 | RB1 | TUSC1 |  |
| BECN1 | CTSB | FOS | ITPR1 | NPC1 | RB1CC1 | ULK1 |  |
