# Supplementary figures and images for: Development and Validation of a Prognostic Signature Associated With Tumor Microenvironment Based on Autophagy-Related lncRNA Analysis in Hepatocellular Carcinoma
Source: Front Med (Lausanne). 2021 Dec 14;8:762570. doi: 10.3389/fmed.2021.762570 (PMC8712323; doi:10.3389/fmed.2021.762570)

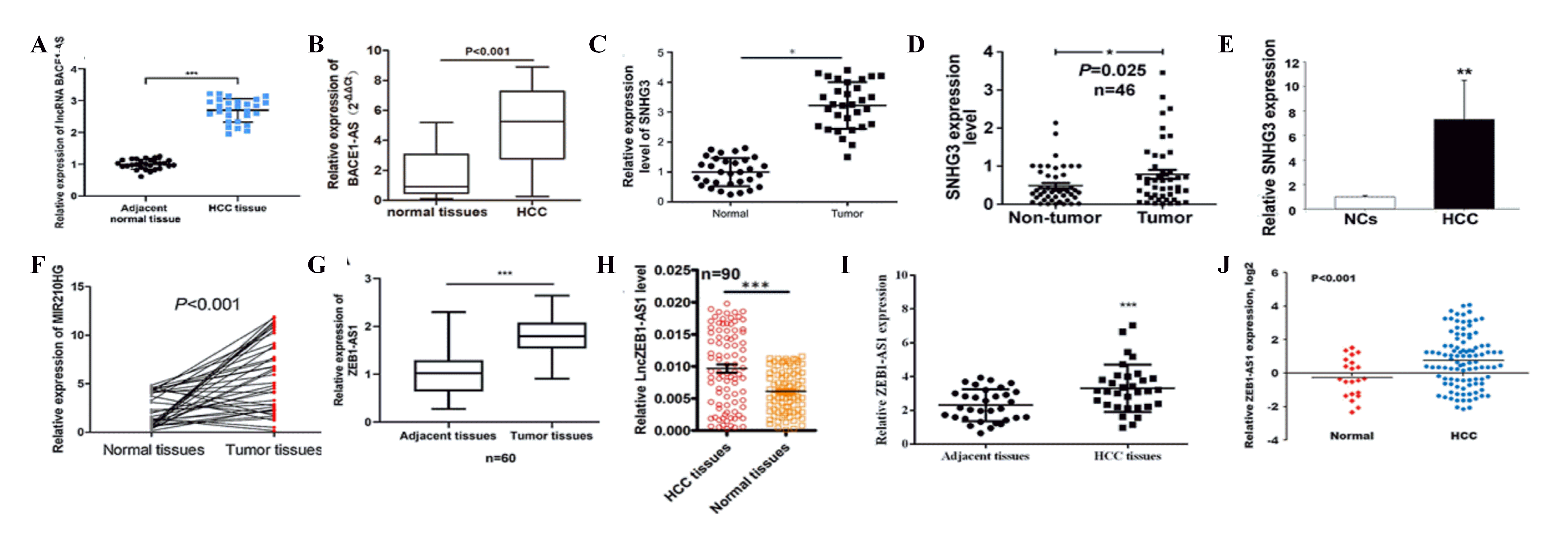

Supplement: Supplementary Figure S1 — The expression levels of the four autophagy-related long non-coding RNAs (lncRNAs) in hepatocellular carcinoma (HCC) tissues and adjacent normal tissues were detected by RT-PCR in each previous study. BACE1-AS (A–B), SNHG3 (C–E), MIR210HG (F), and ZEB1-AS1 (G–J). [file Image_1.TIF]
